# Neuromodulatory Inputs to Motoneurons Contribute to the Loss of Independent Joint Control in Chronic Moderate to Severe Hemiparetic Stroke

**DOI:** 10.3389/fneur.2018.00470

**Published:** 2018-06-21

**Authors:** Jacob G. McPherson, Michael D. Ellis, R. Norman Harden, Carolina Carmona, Justin M. Drogos, Charles J. Heckman, Julius P. A. Dewald

**Affiliations:** ^1^Department of Physical Therapy and Human Movement Sciences, Northwestern University Feinberg School of Medicine, Chicago, IL, United States; ^2^Department of Biomedical Engineering, Florida International University, Miami, FL, United States; ^3^Department of Physiology, Northwestern University Feinberg School of Medicine, Chicago, IL, United States; ^4^Department of Physical Medicine and Rehabilitation, Northwestern University Feinberg School of Medicine, Chicago, IL, United States; ^5^Department of Biomedical Engineering, Northwestern University, Evanston, IL, United States

**Keywords:** stroke, norepinephrine, motoneuron, motor impairment, rehabilitation, brainstem, motor control

## Abstract

In chronic hemiparetic stroke, increased shoulder abductor activity causes involuntary increases in elbow, wrist, and finger flexor activation, an abnormal muscle coactivation pattern known as the flexion synergy. Recent evidence suggests that flexion synergy expression may reflect recruitment of contralesional cortico-reticulospinal motor pathways following damage to the ipsilesional corticospinal tract. However, because reticulospinal motor pathways produce relatively weak post-synaptic potentials in motoneurons, it is unknown how preferential use of these pathways could lead to robust muscle activation. Here, we hypothesize that the descending neuromodulatory component of the ponto-medullary reticular formation, which uses the monoaminergic neurotransmitters norepinephrine and serotonin, serves as a gain control mechanism to facilitate motoneuron responses to reticulospinal motor commands. Thus, inhibition of the neuromodulatory component would reduce flexion synergy expression by disfacilitating spinal motoneurons. To test this hypothesis, we conducted a pre-clinical study utilizing two targeted neuropharmacological probes and inert placebo in a cohort of 16 individuals with chronic hemiparetic stroke. Test compounds included Tizanidine (TIZ), a noradrenergic α_2_ agonist and imidazoline ligand selected for its ability to reduce descending noradrenergic drive, and Isradipine, a dihyropyridine calcium-channel antagonist selected for its ability to post-synaptically mitigate a portion of the excitatory effects of monoamines on motoneurons. We used a previously validated robotic measure to quantify flexion synergy expression. We found that Tizanidine significantly reduced expression of the flexion synergy. A predominantly spinal action for this effect is unlikely because Tizanidine is an agonist acting on a baseline of spinal noradrenergic drive that is likely to be pathologically enhanced post-stroke due to increased reliance on cortico-reticulospinal motor pathways. Although spinal actions of TIZ cannot be excluded, particularly from Group II pathways, our finding is consistent with a supraspinal action of Tizanidine to reduce descending noradrenergic drive and disfacilitate motoneurons. The effects of Isradipine were not different from placebo, likely related to poor central bioavailability. These results support the hypothesis that the descending monoaminergic component of the ponto-medullary reticular formation plays a key role in flexion synergy expression in chronic hemiparetic stroke. These results may provide the basis for new therapeutic strategies to complement physical rehabilitation.

## Introduction

In chronic hemiparetic stroke, the ability to perform precise, single-joint movements with the paretic arm is often replaced by the constraint to patterns of grouped, multi-joint movements such as the “flexion synergy” (1, 2). The underlying impairment leading to flexion synergy expression can be characterized both mechanically and electromyographically (3) by quantifying abnormal coupling of distal joint torques (4–6) and distal flexor muscle activation (6–9), respectively, during abduction loading/deltoid activation. However, quantifying the ability to move against the constraints imposed by expression of the flexion synergy (9–12) affords greater insight into the individual's loss of independent joint control (13), arguably the primary source of reaching dysfunction. Although the neural mechanisms underlying the flexion synergy expression remain incompletely understood, recent evidence suggests that it is related to progressive recruitment of contralesional cortico-reticulospinal motor tracts as the requirements for descending neural drive to the paretic arm exceed the capabilities of the spared ipsilesional motor resources (14).

An important, unanswered question regarding recruitment of cortico-reticulospinal pathways is how the relatively weak post-synaptic potentials generated by reticulospinal motor axons could lead to robust muscle activation. Indeed, the magnitude of reticulospinal post-synaptic potentials in spinal α-motoneurons is only ~20% as great as that of post-synaptic potentials elicited by intact corticospinal axons (15). Here, we hypothesize that reticulospinal post-synaptic potentials are amplified by the descending neuromodulatory component of the ponto-medullary reticular formation (PMRF), which is coactivated with PMRF motor pathways (16–20). The descending neuromodulatory component of the PMRF utilizes the monoamines serotonin (5-HT) and norepinephrine (NE) to regulate the sensitivity of spinal motoneurons and interneurons to ionotropic inputs (21). More specifically, 5-HT and NE markedly increase all aspects of motoneuron excitability—depolarizing the resting membrane potential, hyperpolarizing the spiking threshold, shortening the afterhyperpolarization period, and facilitating persistent inward currents (PICs) (18, 22–25)—while generally inhibiting spinal sensory neurotransmission (26, 27).

If the neuromodulatory component of the PMRF amplifies the motor commands that drive flexion synergy expression, then inhibition of the neuromodulatory component would be predicted to reduce flexion synergy expression by removing this potent source of motoneuron facilitation. To test this prediction, we conducted a preclinical investigation in a cohort of 16 individuals with hemiparetic stroke that utilized two targeted neuropharmacological probes and an established quantitative kinematic/kinetic measure of flexion synergy expression (3, 10, 11). Pharmacological test compounds included Tizanidine hydrochloride (TIZ), a noradrenergic α_2_ agonist and imidazoline ligand (28, 29); Isradipine (ISR), a dihydropyridine calcium channel antagonist (30, 31); and inert placebo (PLC). TIZ has two potential sites/mechanisms of action in this context: spinal, where it inhibits polysynaptic group II sensory input to motoneurons (32, 33), and supraspinal, where it reduces NE release into the spinal cord via a combination of effects on inhibitory noradrenergic autoreceptors and on imidazoline I_3_ receptors in the PMRF (28, 29, 34–36). ISR is expected to have only a spinal site/mechanism of action, where it blocks the Ca_v_1.3 channels required for NE to amplify post-synaptic potentials in motoneurons via PICs (37, 38).

We found that TIZ, but not ISR or placebo, was capable of significantly decreasing the impact of flexion synergy expression on reaching work area post-stroke. This finding is consistent with a supraspinal action of TIZ to reduce the release of NE in the spinal cord: if stroke induces an increase in monoaminergic output from the brainstem, then spinal sensory input should have already been suppressed (due to increased central NE) and the addition of TIZ should have little effect on spinal sensory pathways. In contrast, the autoreceptor/imidazoline mechanism of TIZ opposes rather than mimics brainstem monoaminergic output, and is thus more likely to have a substantial impact on motor function if indeed monoaminergic drive is increased post-stroke. In either scenario, however, this finding supports the hypothesis that the descending neuromodulatory component of the PMRF plays a key role in enabling vigorous expression of the flexion synergy. This finding also provides compelling evidence for the progression toward early phase clinical trials investigating both the independent effects of TIZ on motor function and the augmentative effects of TIZ in combination with physical interventions designed to target flexion synergy (39, 40).

## Methods

### Participant recruitment, retention, and clinical data

This investigation was approved by the Institutional Review Board of Northwestern University in accordance with the ethical standards stipulated by the 1964 Declaration of Helsinki for research involving human participants. All potential participants provided informed, written consent to participate. Experiments and clinical examinations were performed in dedicated laboratory and outpatient clinical space in an academic medical center.

Each potential participant was screened by the research physical therapist and research physician to determine if they satisfied the eligibility criteria. For inclusion in the study, participants were required to meet all of the following criteria: first-ever unilateral brain injury due to stroke at least 1 year prior to enrollment in the study; paresis confined to the contralesional side of the body, with motor impairment of the arm; absence of muscle tone abnormalities and motor or sensory impairments in the ipsilesional arm; absence of severe wasting or contracture of the paretic arm; intact/impaired but not absent sensation in the paretic arm; ability to perform a 3-step command; absence of severe concurrent medical problems (e.g., cardiorespiratory impairment); absence of brainstem lesions, as determined from clinical and radiological reports; no current or previous history of untreated or active renal, liver, respiratory, or cardiac impairments; no concurrent use of medications known to suppress central nervous system activity, including alcohol; at least 90° of passive range of motion for both shoulder flexion (horizontal adduction) and shoulder abduction in the paretic arm; and some volitional control of shoulder abduction and elbow flexion/extension. Recent changes in the medical management of hypertension, current use of anti-spastic medications (e.g., baclofen or botulinum toxins), or contraindication for using either test compound were grounds for exclusion from the study. Eligibility criteria were not changed after the study enrollment period began.

Of the 441 individuals whom agreed to be contacted regarding potential participation in the study, 375 were interviewed via phone call to assess potential admissibility. Of those, 116 were screened in-person by the research physical therapist, with 27 eligible individuals subsequently screened by the research physician. Twenty of these individuals fulfilled all criteria for participation and were enrolled, randomized, and began the study. Sixteen individuals completed at least one full phase of the investigation (i.e., pre-drug, on-drug, and washout) for an active test compound (not placebo) and are included in the analyses presented herein (mean age: 59 ± 12 years; Table 1); 15 individuals completed all three phases of the investigation (i.e., TIZ, ISR, PLC). This number of completed participants met our minimum sample size requirement (*N* = 15), which was determined *a priori* based on effect sizes derived from preliminary data as well as a desired power of 0.9 and α = 0.05.

**Table 1:** Participant demographic and clinical data.

**Participant Code**	**Age**	**Biological sex**	**Years post-stroke**	**Affected limb**	**Lesion location**	**Upper limb Fugl-Meyer motor assessment**	**Affected limb hand dynamometry**
A	51	M	2	L	Large R cerebral hemispheric lesion; frontal temporal, parietal, and occipital lobes (cortex and white matter)	21/66	4.6 Kg
B	63	F	4	R	Not available	15/66	6.0 Kg
C	62	F	7	R	L thal., int. cap, BG, putamen, GP, lentiform nucleus	16/66	4.0 Kg
D	81	M	5	L	Not available	37/66	18.6 Kg
E	60	M	3	R	L cortical and subcortical limbic cortex, int. cap.	43/66	26.0Kg
F	61	M	2	R	L BG, int. cap., insular cortex, sup./inf. frontal gyrus and subcortical white matter	15/66	10.6 Kg
G	41	M	6	L	R int. cap., R BG, R thal.	38/66	10.0 Kg
H	62	F	24	R	L putamen, GP, L thal., int. cap.	12/66	6.2 Kg
I	65	M	10	R	L parietal lobe, insular cortex, lentiform nucleus, thal., genu and post.-limb int. cap., external and extreme capsule, distention of L lat. Ventricle	20/66	9.3 Kg
J	55	M	4	L	R BG, int. cap.	25/66	10.6 Kg
K	51	M	4	R	Not available	14/66	6.0 Kg
L	63	M	8	L	Cortical lesion: R sup/mid/inf frontal gyri, int. cap., thal.	35/66	6.6 Kg
M	30	F	6	L	Not available	44/66	14.6 Kg
N	60	M	4	L	R posterior frontal cortex (premotor and motor)	45/66	22.0 Kg
O	57	M	1	L	R lacunar infarct at genu of int. cap.	16/66	4.3 Kg
P	67	M	6	L	Not available	12/66	9.0 Kg

*Age, years post-stroke, side of hemiparesis (i.e., affected limb), lesion location, Fugl-Meyer Motor Assessment score, hand dynamometry of affected limb. Lesion location abbreviations: thal, thalamus; int. cap.; internal capsule; BG, basal ganglia; GP, globus pallidus*.

With the exception of one participant, all individuals for whom specific lesion data were available exhibited white matter damage to the internal capsule as a component of the infarct (Table 1). In the participant without capsular involvement, pre-motor and motor cortical sites were affected. Upper limb Fugl-Meyer Motor Assessment (FMA) scores ranged from 12 to 45 of a possible 66 across participants, representing severe to moderate impairment (Table 1).

### Study design, randomization, and blinding

This pre-clinical study employed a randomized, double-blind, placebo-controlled crossover design in which each participant was sequentially administered each test compound in a randomized order. Given the crossover design, the study was completely randomized (i.e., no blocking) and participant allocation ratio was inherently equal for each drug (and placebo). Randomization sequences were computer generated by the research pharmacist and were determined for the entire cohort prior to enrollment of any participants. We administered all test compounds chronically rather than using a single-dose design. Although our approach more accurately mirrors a potential clinical use scenario, we chose this approach to control for the adaptation period of monoaminergic drugs, during which their effects may change.

Study participants and experimentalists, including the research physician and research physical therapist, were blind to the test compound being administered at all times. The blind was achieved by overencapsulation of the medications into identical capsules. Test compounds were delivered three times a day to accommodate the shorter half-life of TIZ, and placebo capsules were used to blind the dosing scheme during periods when participants were being administered ISR, the longer half-life drug. The research pharmacist maintained the blind, and the unblinding key was located in a locked office not used for data collection or clinical examination. The research pharmacist was not involved in recruitment, consent, data collection, clinical examination, analysis, or interpretation of findings. The study was considered complete and the blind was broken after the entire participant cohort had completed the study and all results were analyzed.

Participant safety monitoring and medical data quality control were performed by the research physician and nurse (both blinded), and included weekly phone calls as well as routine office visits. Laboratory data monitoring and quality control were performed by members of the research team, who were also blinded. An additional, external data safety and monitoring board was not included for this investigation, per the current guidelines established by the National Institutes of Health for low-risk, single-site, early phase investigations (NIH Notice: OD-00-038).

### Participant flow through study

**Figure 1:**
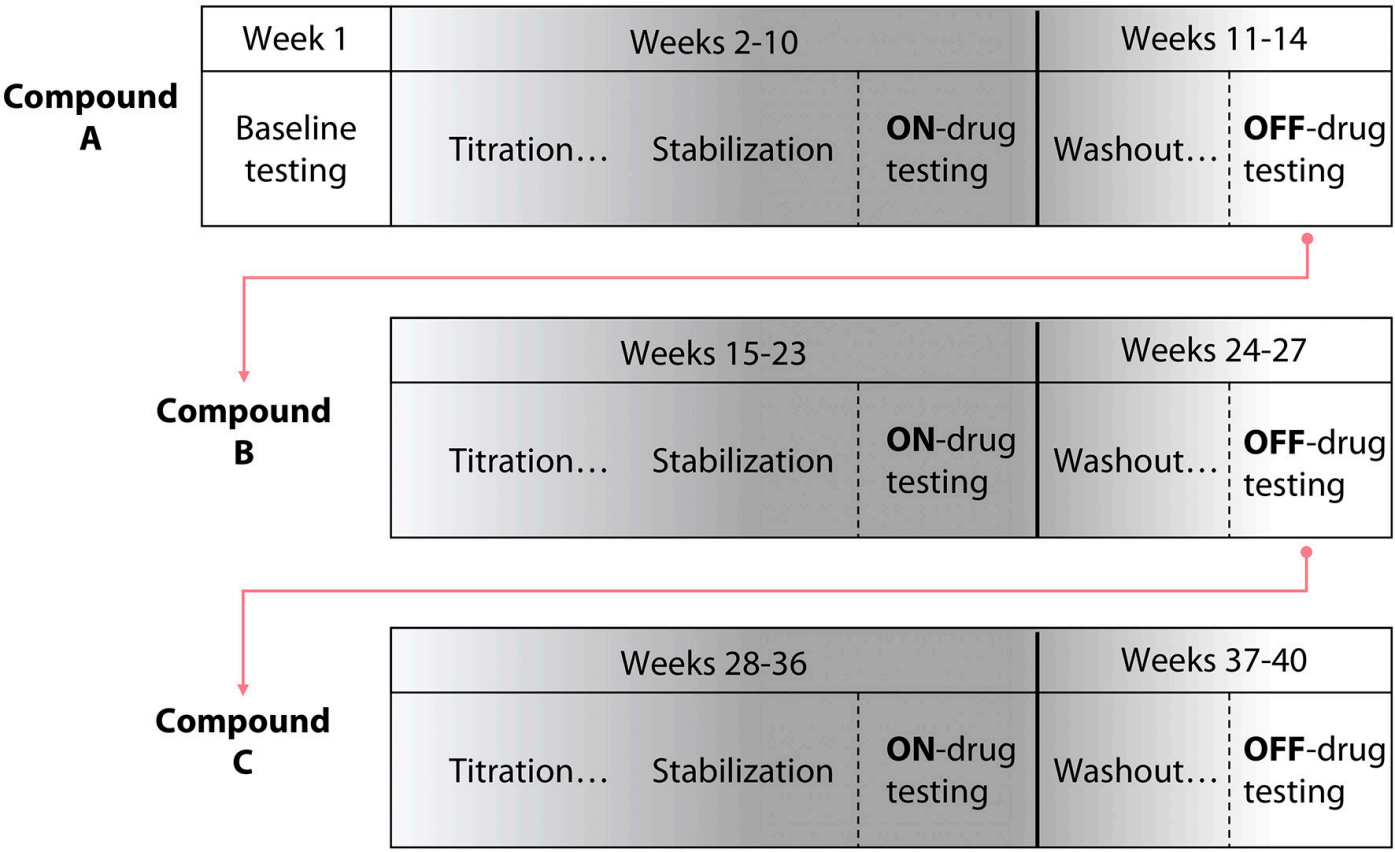
Study design schematic. A randomized, double blind, placebo-controlled crossover pre-clinical study was conducted using three test compounds. The design included 5 weeks of titration with each test compound, 4 weeks of stabilization, and 4 weeks of washout. The template for a participant's progression through the study is shown; test compound administration order was randomized.

The study consisted of three phases, each associated with a different test compound (Tizanidine, Isradipine, or Placebo; Figure 1). Each phase progressed as follows: maximum voluntary strength and the impact of flexion synergy expression on reaching performance were initially tested on week one. Subsequently, each participant was titrated to the recommended therapeutic or maximum tolerable dose of test compound over the course of 5 weeks. Participants were then allowed to stabilize at pharmacological steady-state for a period of 4 weeks. During the 4th week of stabilization (9th week of test compound administration), maximum strength and the impact of flexion synergy expression on reaching performance were reassessed. Finally, participants were washed out of test compound over the course of 4 additional weeks. In the 4th week of washout, maximum strength, and the impact of flexion synergy expression on reaching performance were tested again. This sequence was repeated for the remaining test compounds (Figure 1). Total study duration per participant was therefore ~9 months.

### Test compounds, titration schedule

**Figure 2:**
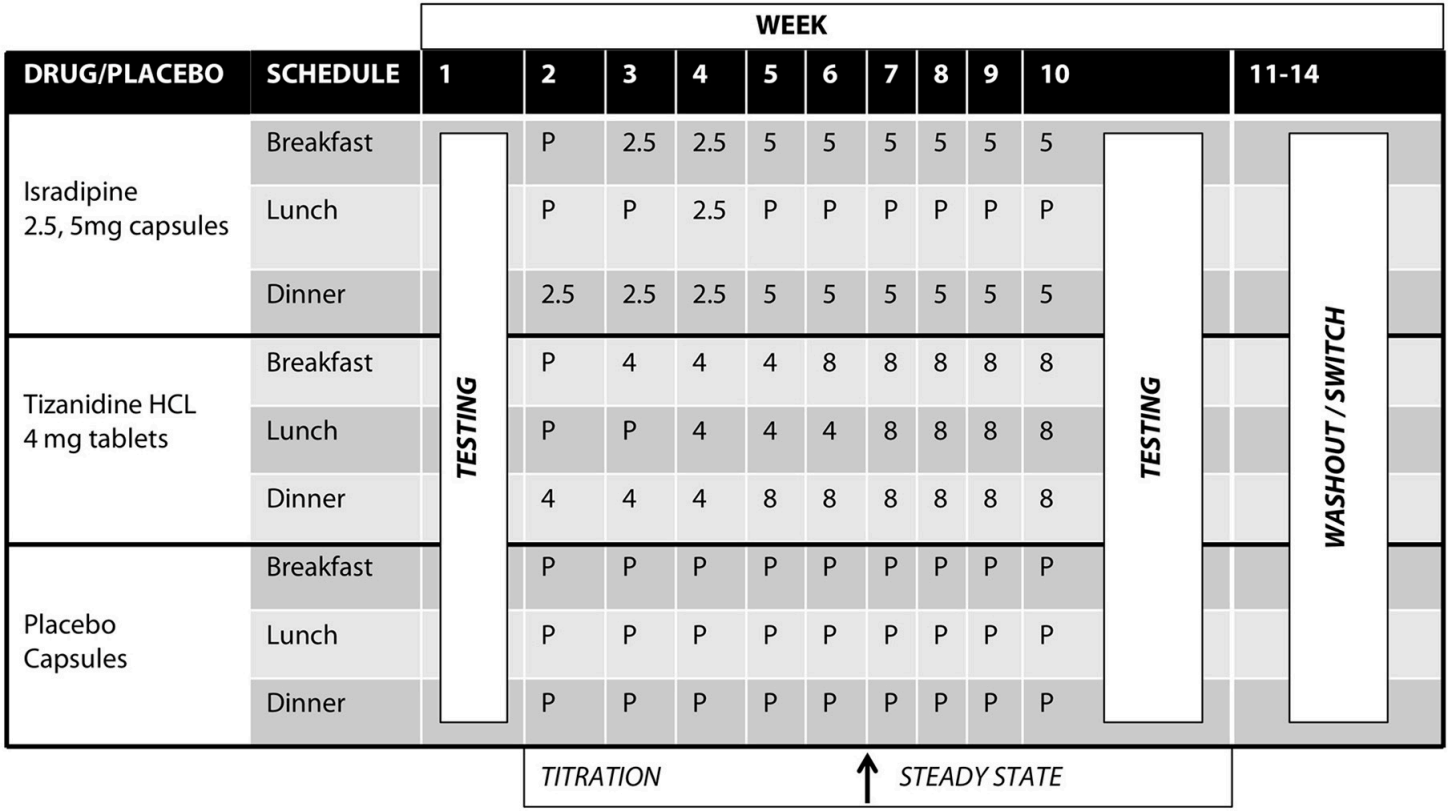
Test compound titration schedule. Isradipine was administered at 2.5 mg/day escalating to 10 mg/day over the course of 5 weeks; Tizanidine was initially administered at 4 mg/day, escalating to 24 mg/day over the course of 5 weeks. After titration to maximal dosage, participants were allowed to stabilize for an additional 4 weeks. All test compounds were over-encapsulated to maintain the study blind.

Tizanidine hydrochloride (TIZ) is a centrally acting noradrenergic α_2_ agonist that contains an imidazoline ring and is structurally related to clonidine. TIZ is currently indicated for the management of spasticity secondary to multiple sclerosis, spinal cord injury, and stroke. TIZ is known to agonize inhibitory noradrenergic α_2_ auto-receptors located on descending coeruleospinal projections (34, 36, 41). TIZ is also active at imidazoline binding sites (28, 29, 42, 43), and pharmacological manipulations utilizing TIZ in addition to yohimbine, idazoxan, efaroxan, and RX821002 implicated the I_3_ site in particular (28). TIZ was initially administered at 4 mg/day in the evening, escalating to 8 mg three times daily (24 mg/day total) or maximum tolerable dose over the course of 5 weeks (Figure 2). The normal range of dosing for TIZ is 4 mg every 8 h (12 mg/day, min) up to 12 mg every 8 h (36 mg/day, max).

Isradipine (ISR) is a dihydropyridine calcium channel antagonist capable of penetrating the blood brain barrier (30). Clinically, ISR is indicated for the management of hypertension. ISR was initially administered as 2.5 mg in the evening, escalating to 5 mg twice daily (10 mg/day total) (Figure 2). Normal dosing for ISR is 2.5 to 5 mg twice daily (typical therapeutic dose is 10 mg/day, though tolerated to 20 mg/day max).

### Flexion synergy assessment, experimental protocol

Using a previously described robotic device, the ACT-3D system (10), the impact of flexion synergy expression on horizontal reaching work area was measured prior to and during the administration of each test compound. Quantification of reaching work area (10) is a robust and validated evaluation of the impact of flexion synergy expression on reaching function (11). Briefly, the ACT-3D consists of an admittance-controlled HapticMaster robot retrofitted with a 6 degrees of freedom loadcell and instrumented gimball (Moog-FCS, The Netherlands), a Biodex experimental chair (Biodex Medical Systems, Shirley, NY) and a custom graphical user interface. The HapticMaster allows unrestrained motion of the upper limb in three dimensions, and can impose force or position disturbances upon its user. Its graphical user interface displays an avatar of the participant's arm, which moves in real time with actual limb displacement.

The following protocol was performed in its entirety at each experimental visit during the study. First, we collected maximum voluntary torques (MVTs) for shoulder abduction, elbow extension and elbow flexion torques in the paretic arm, as previously described (44). Next, we interfaced participants with the ACT-3D robotic device for flexion synergy assessments. Participants were secured in and secured to the Biodex experimental chair of the ACT-3D via over-the-shoulder and lap-belt restraints. Subsequently, the forearm, wrist and hand of the participant's paretic limb were placed in a rigid orthosis mounted via revolute joint to the end effector of the HapticMaster robot. After placement of the arm into the orthosis, the height of the chair was adjusted such that the participant exhibited 90° of shoulder abduction, which was maintained throughout the experiment.

To measure the impact of flexion synergy reaching performance, participants were required to generate the largest horizontal reaching work area (i.e., largest circles) possible with the paretic limb either while moving over a frictionless virtual table surface simulated by the ACT-3D or while lifting the arm from this virtual surface. If instructed to lift, participants were subjected to a vertical force from the ACT-3D, causing the limb to feel lighter or heavier than normal; that is, the force applied to the limb by the ACT-3D altered the amount of shoulder abduction torque the participant was required to generate during work area measurement. Shoulder abduction torque requirements were normalized to each individual's abduction strength, such that all participants generated work areas at four standardized levels. These levels included moving over the aforementioned frictionless table (termed “Table”), lifting yet generating no net shoulder abduction torque (i.e., the weight of the limb was fully compensated by the ACT-3D; the limb felt as if it was floating), lifting while producing 25% of abduction MVT, and lifting while producing 50% of abduction MVT. All work areas were generated at elbow angular velocities of <10°/s in order to avoid the influence of stretch-sensitive reflexes. It should also be noted that the apparent inertia of the limb did not change as shoulder abduction loading was increased nor did the required abduction torque change anywhere in the horizontal workspace. A minimum of four trials was collected at each load level; each trial involved generation of ~2–3 work areas, and trials were collected in both the clockwise and counter-clockwise directions.

### Data analysis

Custom Matlab software (The MathWorks, Natick, MA) was used for all data analysis. Individual work area trials at a given shoulder abduction load level were overlaid and the maximum perimeter was extracted; the area enclosed by this perimeter reflected the largest work area (in square meters) accessible by the participant at that shoulder abduction load level. To account for the anthropometric differences between participants, each participant's work area values were normalized to the work area obtained while supported on the haptic table. The difference in work area from pre- to on-drug was computed at each load level, expressed as a percentage of the work area obtained while supported on the haptic table, and these values were saved for statistical analysis.

#### Statistical analysis

We used a linear mixed model to evaluate the potential effect of test compound administration on the impact of flexion synergy expression during planar reaching, our primary outcome measure. Independent variables in the model included participant (1–16), experimental phase, test compound, and shoulder abduction load level. The dependent variable in the model was the difference between reaching work area before administration of each test compound compared to reaching work area during test compound administration (which, to reiterate, was expressed as a percentage of the maximum work area generated in the “table” condition). Participant was included in the model as a random factor, while experimental phase, test compound, and shoulder abduction load level were repeated fixed effects. The experimental phase parameter was an integer value ranging from 1 to 3 that reflected the literal phase of the study a participant was in at a given time. For all participants, the experimental phase parameter was in the order “1, 2, 3,” regardless of the test compound being administered during that sequence. Experimental phase was expected to be non-significant and was included in the model to test the effectiveness of the drug order randomization. The test compound parameter had values of Tizanidine (TIZ), Isradipine (ISR), or placebo (PLC). The shoulder abduction load level parameter reflected the amount of shoulder abduction torque being generated during the work area measurement; this variable had four levels, including “Table,” “Floating” (0% SABD MVT), 25 and 50% SABD MVT. The model's dependent variable, difference between reaching work area before administration of each test compound compared to reaching work area during test compound administration, was computed at each shoulder abduction load level. A compound symmetry covariance structure was assumed for fixed and random effects. Post-tests were decided *a priori*, and included change in work area with ISR compared to that of PLC, and change in work area with TIZ compared to that of PLC.

Secondary analyses included the potential impact of test compound administration on maximum strength, the stability of work area measurements over time (i.e., to test for changes in reaching performance not associated with study interventions), and the effectiveness of our washout periods. As appropriate, normality was confirmed *a priori* with the Shapiro-Wilk test and the assumption of sphericity was assessed using Mauchly's test. Two-tailed paired *t*-tests were performed across participants to compare MVTs produced before test compound administration to those generated while fully titrated and stabilized on each test compound. A two-way repeated measures ANOVA with factors of period and support level and a dependent variable of work area was used to assess the stability of baseline work area measures over time, and a two-way repeated measures ANOVA was used to evaluate the effectiveness of the washout periods, with factors of “previously administered medication,” and shoulder abduction load level.

Bonferroni correction was used to control the family-wise error rate, and a *P*-value of 0.05 or less was considered significant in all cases. No changes were made to outcome measures after enrollment began. All statistical analyses were performed using IMB SPSS software (version 19).

## Results

### Primary outcome measure

**Figure 3:**
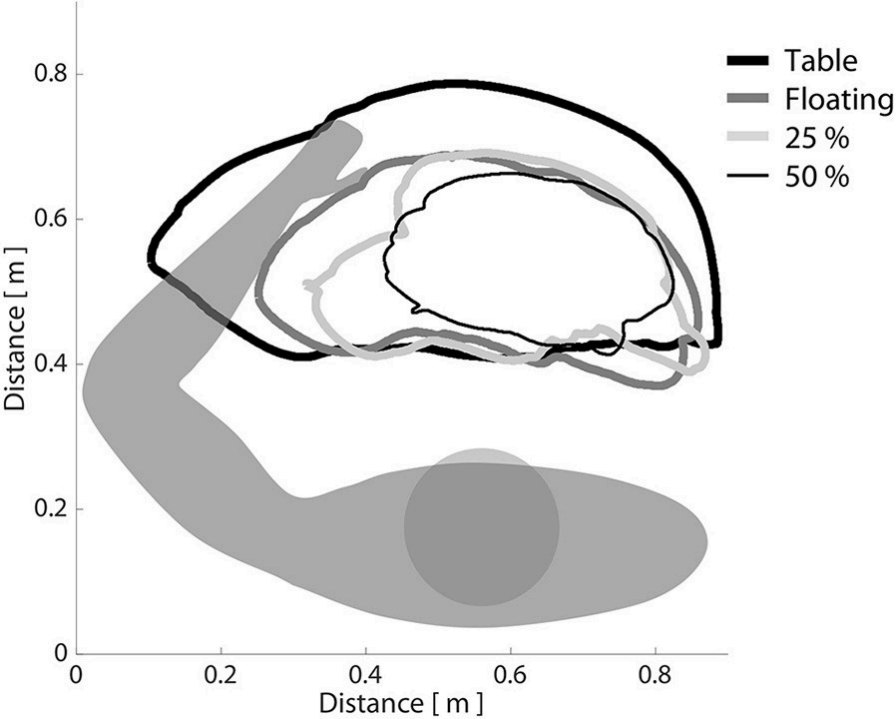
The effect of shoulder abduction loading on paretic limb work area. Participants were asked to generate the largest horizontal reaching work area possible with the paretic limb, while a robotic device manipulated shoulder abduction load level. It can be seen that as shoulder abduction loading increased, work area decreased, consistent with the definition of the flexion synergy. Bold black line: moving over a frictionless virtual table; bold dark gray line: limb fully supported by ACT-3D, as if weightless; light gray line: generating 25% of maximum voluntary shoulder abduction torque; narrow black line: generating 50% of maximum voluntary shoulder abduction torque.

Using data extracted from the initial baseline testing session, we first confirmed across all participants that increasing shoulder abduction torque led to progressive decreases in horizontal reaching work area with the paretic arm. This trend can be seen in Figure 3, in which a single, representative participant with moderate impairment (FMA: 24/66) is required to produce progressively increasing amounts of shoulder abduction torque while generating the largest circles possible with the paretic arm. Across all participants, work area (expressed as a percentage of on-table area) averaged 69.5% when lifting and producing 0% abduction MVT (limb made to feel weightless, as if floating), 56.5% when lifting and producing 25% abduction MVT, and 45.1% when lifting and producing 50% abduction MVT. It should be reiterated that no net abduction torque was required to complete the task in either the on-table condition or the 0% abduction MVT condition. Nevertheless, work area was ~30% smaller in the 0% abduction MVT condition. This finding is presumably related to the ability of individuals to generate shoulder adduction torque when moving over the haptic table, which may facilitate elbow extension due to expression of the extension synergy.

**Figure 4:**
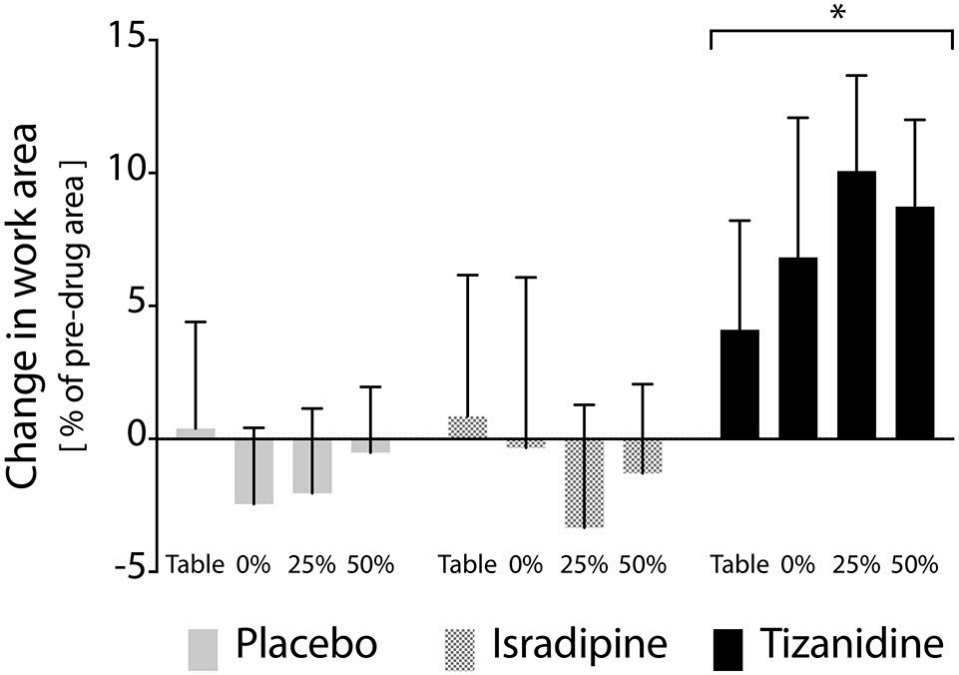
Effects of test compound administration on reaching work area. Solid gray bars: placebo, PLC; Hatched gray bars: Isradipine, ISR; Black bars: Tizanidine, TIZ. Y-axis (bar height) indicates change in work area from pre-administration measures to those recorded when stabilized at maximum dosage, expressed as a percentage of maximum work area; positive changes indicate an increase in work area during test compound administration. Error bars: 95% confidence interval. X-axis: shoulder abduction load condition (percentages are of MVT). Across shoulder abduction load levels, chronic administration of TIZ led to a significant increase in work area as compared to administration of PLC; ISR was not different than PLC. ^*^Significant difference relative to placebo.

After all participants had completed each phase of the study, we examined the results of our linear mixed model exploring the primary outcome measure of test compound administration on reaching work area. Across the overall cohort of 16 participants, 14 participants completed the ISR sequence, and 13 participants each completed the TIZ and PLC sequences. All available data were used in the linear mixed model. Expectedly, the experimental phase factor did not achieve significance at the alpha = 0.05 level (*F* = 0.209, *P* = 0.812). This finding confirms that randomization of test compound order between participants was effective. The treatment factor did achieve significance, however (*F* = 3.865, *P* = 0.023), indicating that administration of at least one test compound affected a change in work area as compared to its corresponding pre-drug measure. *Post-hoc* analysis revealed that the difference in work area from pre-TIZ to on-TIZ was significantly greater than the difference observed before and during administration of PLC (*P* = 0.026), while the difference in work area from pre-ISR to on-ISR was not different than the difference in work area observed before and during administration of PLC (*P* = 1.0), (Figure 4). The statistical model revealed no significant effect of support level (*F* = 0.112, *P* = 0.953) on the impact of test compound on work area, indicating that changes in work area from before test compound administration to during administration were uniform across abduction load levels (although a non-significant decrease in work area percent change was observed when lifting at 50% abduction MVT during TIZ administration). Average differences in work area from before to during test compound administration (averaged across all abduction load levels) were as follows: ISR: −1.0%, TIZ: +7.4%, PLC: −0.8% (negative values reflect a reduction in work area during test compound administration; positive values reflect an increase in work area during drug administration; Figure 4).

### Secondary outcome measures

**Figure 5:**
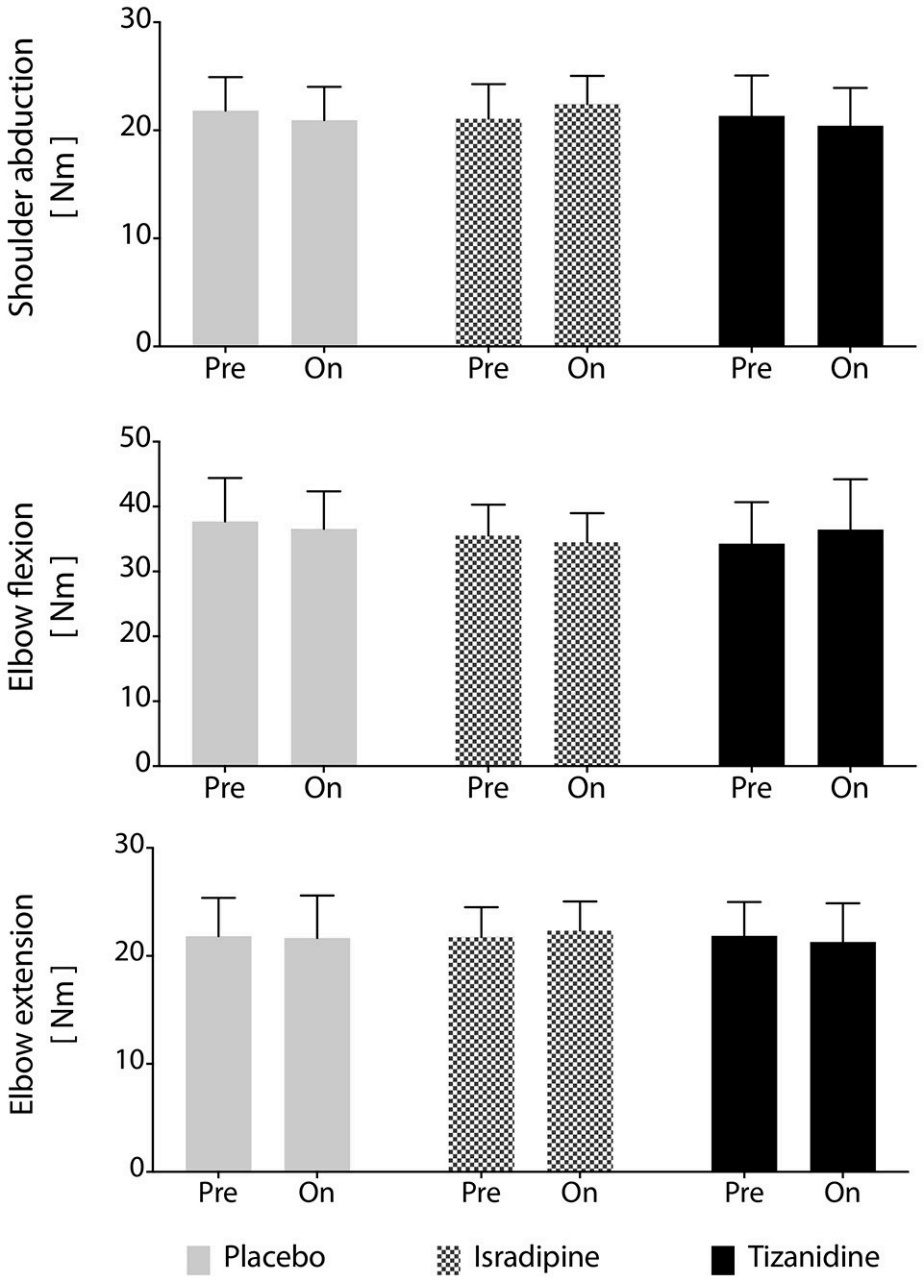
Maximum voluntary torque production is unchanged by test compound administration. Average maximum voluntary torque in shoulder abduction, elbow flexion, and elbow extension across participants (*N* = 16). No significant differences were found between maximum voluntary torques generated during administration of any test compound or during the washout periods. Solid gray bars: placebo; hatched gray bars: Isradipine; Black bars: Tizanidine.

Secondary outcome measures included maximum strength, stability of the work area measurement over time, and effectiveness of the washout periods. We found no changes in MVT magnitude in elbow extension, elbow flexion, or shoulder abduction prior to administration of any test compound or at any during-administration time point (Figure 5). Given that each participant's involvement in the study required 7 testing sequences over the course of 9 months, it was of interest to know whether work area was a stable metric over the time course of the investigation. To assess the stability of our work area metric over time, we compared work areas measured before administration of each participant's first test compound to work areas measured prior to administration of the second and third test compounds (after washout), regardless of the order of test compounds administered to each participant. We found no significant effect of experiment phase on work area measurement (*P* = 0.463), confirming the robustness of the work area metric over time. Finally, we assessed the effectiveness of the 4-week washout periods after each test compound administration. For washout periods to be considered successful, work areas measured during each washout period should not differ, regardless of the test compound previously administered. To test this hypothesis, each participant's data was re-aligned by test compound such that post-ISR work areas were compared to post-TIZ and post-PLC areas. We found that work areas generated at the conclusion of washout did not differ based on the test compound previously administered (*P* = 0.537), indicating a lack of “carry-over” effects of either drug or placebo.

## Discussion

Expression of the flexion synergy is thought to arise through progressive recruitment of contralesional cortico-reticulospinal motor pathways (14). Here, we hypothesized that descending monoaminergic pathways from the PMRF are likely to play a critical role in amplifying the relatively weak post-synaptic potentials generated by reticulospinal motor pathways. This would enable vigorous expression of the flexion synergy, constraining reaching function when abducting the shoulder against gravity. Indeed, in the *absence* of monoaminergic neuromodulation, maximum activation of the excitatory components of corticospinal, rubrospinal, and vestibulospinal motor pathways would likely be inadequate to activate a sufficient number of motoneurons to produce vigorous functional contractions (45–47). Thus, we predicted that an overall reduction in descending monoaminergic drive, or a suppression of monoaminergic effects within the spinal cord itself, would reduce constraints imposed by the expression of flexion synergy (and thus improve reaching function) in a manner analogous to reductions in muscle tone during natural sleep or anesthesia-induced atonia (19, 20).

### Tizanidine reduces flexion synergy expression

We found that reaching work area increased by an average of 7.4% across shoulder abduction load levels during TIZ administration. We interpret this finding as most likely related to a TIZ-mediated disfacilitation of motoneurons via effects on coeruleospinal projections and/or effects on imidazolinergic receptors in the PMRF. Indeed, TIZ agonizes inhibitory α_2_ autoreceptors located on coeruleospinal axons (34, 35, 41), which reduces descending noradrenergic drive (34) by limiting norepinephrine release at noradrenergic synapses on spinal motoneurons and interneurons (48–50). TIZ is also active at imidazoline binding sites (29, 43), and in particular the I_3_ receptor found throughout the brainstem (28). Administration of TIZ can reduce the spontaneous firing of coeruleospinal neurons via effects at I_3_ receptors (51), which also reduces norepinephrine release in the spinal cord (34). Although mechanistically distinct from its direct effects on noradrenergic receptors, the imidazolinergic actions of TIZ on motor pools is thus similar in principle to that of its noradrenergic effects (although the relative contribution of each pharmacological pathway to the net effect in motoneurons is unknown). Our supraspinal interpretation of the increased work area during TIZ administration is also consistent with animal work demonstrating that TIZ reduces spinal motor output by removing noradrenergic facilitation of motoneurons (28).

However, because we administered TIZ systemically, it is also possible that the observed changes in work area are related to direct effects of TIZ on spinal interneurons. Indeed, it has been hypothesized that TIZ increases presynaptic, reciprocal, and non-reciprocal inhibition by facilitating α_2_ receptors on 1a and 1a/1b interneurons (52). In this scenario, increased inhibition would reduce agonist-antagonist co-activation during reaching, potentially enhancing elbow extension ability during concurrent shoulder abduction loading (i.e., flexion synergy expression) as found here. However, further research has demonstrated that Group 1a and 1a/1b interneurons are not directly impacted by TIZ, and clonidine, which is pharmacologically similar to TIZ, actually depresses Group 1a and 1a/1b interneuron activity (53). It is possible that TIZ-mediated depression of Group II afferent pathways (32, 33) could have contributed to a portion of the observed increases in work area; however, the role of TIZ in functional use of group II pathways has recently been challenged (54). Nevertheless, direct spinal effects of TIZ on reaching work area cannot be ruled out.

Importantly, the increase in work area observed during TIZ administration cannot be explained by an increase in volitional strength or a reduction in spasticity. In fact, we found no changes in maximum voluntary torque production during or after TIZ administration in the shoulder abductors, elbow flexors, or elbow extensors (Figure 5). Although this finding is somewhat surprising given the role of TIZ in reducing noradrenergic drive, it is possible that had we used a higher dose of TIZ, strength disparities may have become evident. Regardless, we would have predicted a reduction in volitional strength during TIZ administration, not an increase. Our findings also cannot be explained by medication-induced changes in spasticity because our slow reaching velocity was below threshold for stretch reflex elicitation. Further, an emerging body of work suggests that the relevance of spasticity to volitional movement/functional use of the contralesional arm is negligible (9, 55, 56).

### Flexion synergy expression unchanged by ISR administration

We used ISR to test the prediction that post-synaptic inhibition of monoaminergic actions on spinal motoneurons would also reduce flexion synergy expression. We chose ISR because of its comparatively high affinity for Ca_v_1.3 channels [~40 times greater than that of the related compound nimodipine (31, 57, 58)], which are essential for amplifying ionotropic inputs to motoneurons via persistent inward currents (PICs) (18, 59–61). Contrary to our prediction, however, the effects of ISR administration on flexion synergy expression were not significantly different from those noted during PLC administration. The most parsimonious explanation for ISR's ineffectiveness may be that the compound's central bioavailability was insufficient at the administered dosage to robustly disfacilitate the involved motoneurons. Indeed, the Ca_v_1.2 channel-mediated effects of ISR on blood pressure limited the maximum dosage that could be safely administered, despite a slow, 5-week titration. Future studies of post-synaptic/spinal modulation of monoaminergic effects in the context of flexion synergy expression is clearly warranted. The reasons for this negative result are unclear, but below we briefly discuss likely possibilities.

The lack of ISR-induced effects on reaching work area could be related to its pharmacological mechanism of action relative to the mechanism by which monoaminergic drive impacts activation of the motor pool. As aforementioned, L-type calcium channels are an integral component of motoneuron PICs (60). We predicted that a reduction in PIC amplitude, ostensibly mediated by ISR-induced antagonism of Ca_v_1.3 channels, would reduce motoneuron output in response to reticulospinal motor commands. However, we were unable to estimate PIC amplitude as part of this investigation. Thus, it is unclear whether administration of ISR actually reduced PICs. It is also possible that in the context of flexion synergy expression, monoaminergic “amplification” of reticulospinal motor commands occurs not directly through modification of the post-synaptic potential itself, but rather through subthreshold depolarization of motoneurons in the pool. Indeed, monoamines also increase motoneuron excitability by depolarizing the resting membrane potential (24), which could lead to recruitment of additional motor units in response to a volley of descending motor commands. Because resting membrane potential is not dictated by L-type calcium channels, however, we would not predict an effect of ISR in this scenario.

The lack of ISR-induced effects on reaching work area could, alternatively, be related to a lower than anticipated overall impact of descending monoaminergic drive during flexion synergy expression. For example, it is possible that reticulospinal motor commands, in concert with the remaining ipsilesional/primary corticospinal drive, do not actually require potent amplification by monoamines at the motoneuron level. If so, then the effects of ISR would be minimal because ISR should not directly impact ionotropic motor pathways (whether reticulospinal or corticospinal) or alter the degree of monoaminergic drive itself. However, our finding that work area increased during TIZ administration strongly suggests that monoaminergic effects are an important component of flexion synergy expression, and argues against an explanation in which their effects are minimal.

### Limitations

Because our test compounds were administered systemically and our assays of monoaminergic actions were necessarily indirect, we cannot unequivocally state that TIZ reduced NE release in the spinal cord nor that the central bioavailability of ISR was inadequate to robustly block Ca_v_1.3 receptors on motoneuron. However, when considered with the known pharmacological profile of TIZ, the structure-function of PMRF motor and neuromodulatory pathways in non-human primates (15, 62–64), recent work in humans showing increased reticulospinal white matter integrity in individuals with severe synergy expression post-stroke (65), and increased recruitment of contralesional cortico-reticulospinal motor pathways during flexion synergy expression (14), our findings suggest that TIZ acted, at least in part, via effects on coeruleospinal pathways.

Despite our slow titration schedule, bothersome side effects such as somnolence did occasionally accompany chronic administration of TIZ. Future development of drugs with a milder side effect profile may allow further elucidation of the exact mechanism underlying the expression of flexion synergy. Similarly, the Ca_v_1.2-mediated effects of ISR on blood pressure also limited the maximum dose at which it could safely be administered. Development of Ca_v_1.3-specific L-type calcium channel blockers, which should have fewer cardiovascular effects, represent a welcome advance (66). Other monoaminergic agents (e.g., serotonergic) may further aid our understanding of the mechanisms of flexion synergy expression and have fewer/more tolerable systemic effects.

### Clinical implications

Although we administered TIZ with the goal of probing the mechanisms of flexion synergy expression, we found that TIZ also significantly improved reaching work area in the paretic arm post-stroke. It is noteworthy that the positive impact of TIZ on work area persisted even as shoulder abduction loading increased to 50% of maximum, because it suggests that mechanism-based pharmacotherapies may improve function over a wide range of exertion levels associated with activities of daily living. Previously, our laboratory has shown that a mechanism-based physical rehabilitation intervention consisting of an 8-week training protocol centered about progressive re-introduction of shoulder abduction loading during reaching could also drive significant increases in work area (39, 67) and reaching distance (40). Considering the apparent functional impact of the present study, advancement of the pharmaceutical approach to early-phase clinical trials is warranted. More so, integration of progressive abduction loading therapy (39, 40) with agents such as TIZ may offer the most efficacious potential.

Regarding neuropharmacological aspects of the study, test compounds used in this study were selected on the basis of their receptor pharmacology and were chosen for research purposes only; the results of this study are not intended as an endorsement of either compound for clinical use. Related to this point, we did not perform dose-response investigations as part of this study, and we do not intend to suggest that our dosages of TIZ or ISR are optimal for therapeutic applications; instead, our results highlight the inferences that can be made regarding the mechanisms of flexion synergy expression by using targeted pharmacological probes. Additional investigations are warranted before any preliminary clinical recommendations for amelioration of reaching function can be made.

## Conclusions

In summary, the results of this study supports the hypothesis that the descending monoaminergic component of the PMRF plays a key role in flexion synergy expression and associated loss of independent joint control in individuals with chronic hemiparetic stroke. These findings motivate additional basic scientific investigations utilizing other monoaminergic compounds (e.g., serotonergic) to further probe the role of descending monoaminergic neuromodulation post-stroke. With continued study, these foundational results may provide the basis for new therapeutic strategies to complement physical rehabilitation.

## Author contributions

CH and JPAD designed and supervised the overall research plan; RH designed pharmacological experiments; JM, ME, CH, and JPAD designed specific experimental protocols; JM, ME, CC, and JMD conducted experiments; JM, ME, CH, and JPAD analyzed data; all authors contributed to the interpretation of data and preparation of the manuscript.

### Conflict of interest statement

The authors declare that the research was conducted in the absence of any commercial or financial relationships that could be construed as a potential conflict of interest.
